# Supplementary material for: Polyketide synthase gene clusters in the ericoid mycorrhizal fungus Oidiodendron maius: genome-wide prediction, expression profiling, and functional characterization of knockout mutants
Source: Front Plant Sci. 2026 Sep 16;17:1924430. doi: 10.3389/fpls.2026.1924430 (PMC13623903; doi:10.3389/fpls.2026.1924430)
Supplement: Supplementary file 1 [file SupplementaryFile1.zip › Supplementary Materials/Supplementary Folder 1.zip!/Oidma1_AssemblyScaffolds_Repeatmasked/knownclusterblast/cluster28/jgi.p_Oidma1_20219_mibig_hits.html]

| MIBiG Protein | Description | MIBiG Cluster | MiBiG Product | % ID | % Coverage | BLAST Score | E-value |
| --- | --- | --- | --- | --- | --- | --- | --- |
| XP\_002384860.1 | NADH-dependent\_flavin\_oxidoreductase,\_putative | BGC0001234\_c2 | 4,4'-piperazine-2,5-diyldimethyl-bis-phenol\_biosynthetic\_gene | 49 | 97.8779840849 | 376 | 3.7e-104 |
| EAL94095.1 | NADPH\_dehydrogenase\_Oye3,\_putative | BGC0000811 | Fumigaclavine\_C\_biosynthetic\_gene\_cluster | 49 | 98.1432360743 | 357 | 2.3e-98 |
| ABM91449.1 | putative\_oxidoreductase | BGC0000348 | Ergovaline\_biosynthetic\_gene\_cluster | 49 | 101.591511936 | 351 | 1.3e-96 |
| ABV57819.1 | putative\_oxidase | BGC0001267 | Lysergic\_acid\_/\_elymoclavine\_biosynthetic\_gene\_cluster | 48 | 98.4084880637 | 342 | 5.9e-94 |
| ABK32277.1 | Amb9 | BGC0000014 | Ambruticin\_biosynthetic\_gene\_cluster | 37 | 101.061007958 | 228 | 1.2e-59 |
| ADE22327.1 | oxidoreductase | BGC0000065 | Galbonolides\_biosynthetic\_gene\_cluster | 38 | 93.6339522546 | 208 | 1.3e-53 |
| ACK77755.1 | putative\_oxidoreductase | BGC0000727 | Auricin\_deoxysugar\_moieties\_biosynthetic\_gene\_cluster | 36 | 95.225464191 | 205 | 1.5e-52 |
| CAH10124.1 | putative\_oxidoreducatse | BGC0000268 | Sch47554\_/\_Sch47555\_biosynthetic\_gene\_cluster | 31 | 92.8381962865 | 151 | 2.5e-36 |
| AFO59868.1 | ChxG | BGC0000175 | Cycloheximide\_/\_actiphenol\_biosynthetic\_gene\_cluster | 33 | 74.801061008 | 139 | 9.9e-33 |
| AEA30247.1 | putative\_oxidoreductase | BGC0000429 | Skyllamycin\_biosynthetic\_gene\_cluster | 30 | 96.0212201592 | 131 | 2.1e-30 |
| EED53478.1 | NADH\_oxidase,\_putative | BGC0001304 | Aflavarin\_biosynthetic\_gene\_cluster | 32 | 93.899204244 | 128 | 1.8e-29 |
| ABI91463.1 | NADH:flavin\_oxidoreductase/NADH\_oxidase | BGC0001094 | Enacyloxin\_biosynthetic\_gene\_cluster | 35 | 57.2944297082 | 118 | 1.8e-26 |
| AHH25582.1 | 2,4-dienoyl-CoA\_reductase | BGC0000957 | Ansatrienin\_(mycotrienin)\_biosynthetic\_gene\_cluster | 32 | 55.7029177719 | 99 | 8.7e-21 |
| AAQ84161.1 | PlmM | BGC0000123 | Phoslactomycin\_B\_biosynthetic\_gene\_cluster | 34 | 57.5596816976 | 95 | 1.6e-19 |
| AAM12914.1 | MupC | BGC0000182 | Mupirocin\_biosynthetic\_gene\_cluster | 28 | 81.1671087533 | 86 | 7.6e-17 |
